# The impact of road safety strategy and policy on admissions to a national rehabilitation hospital; a 5-year retrospective review and reflection on trauma data

**DOI:** 10.1186/s12913-023-09177-1

**Published:** 2023-02-21

**Authors:** Áine Carroll, Prasanth Sukumar, Aisling O’Keeffe

**Affiliations:** 1 School of Medicine, University College Dublin, D04 V1W8 Belfield, Dublin, Ireland; 2 National Rehabilitation University Hospital, Rochestown Avenue, Dun Laoghaire, A96 E2H2 Dublin, Ireland

**Keywords:** Road safety strategy, Road Traffic Collision, Rehabilitation, Traumatic brain Injury, Traumatic spinal cord Injury, Trauma system, Major trauma, Retrospective review

## Abstract

**Background:**

Globally, road traffic collisions (RTCs) are a common cause of death and disability. Although many countries, including Ireland, have road safety and trauma strategies, the impact on rehabilitation services is unclear. This study explores how admissions with RTC related injuries to a rehabilitation facility has changed over 5 years and how they contrast to major trauma audit (MTA) serious injury data from the same timeframe.

**Methods:**

A retrospective review of healthcare records with data abstraction in accordance with best practice was performed. Fisher’s exact test and binary logistic regression were used to determine associations and statistical process control was used to analyse variation. All patients discharged with an International Classification of Diseases (ICD) 10 coded diagnosis of Transport accidents from 2014 to 2018 were included. In addition, serious injury data was abstracted from MTA reports.

**Results:**

338 cases were identified. Of these, 173 did not meet the inclusion criteria (readmissions) and were excluded. The total number analyzed was 165. Of these, 121 (73%) were male and 44 (27%) were female and 115 (72%) were under 40 years of age. The majority [128 (78%)] had traumatic brain injuries (TBI), 33 (20%) had traumatic spinal cord injuries and 4 (2.4%) had traumatic amputation The numbers varied over the time period of the study but showed normal variation and not special cause variation which suggests no significant impact of policy in the time frame. There was a large discrepancy between the number of severe TBIs reported in the MTA reports and the numbers admitted with RTC related TBI to the National Rehabilitation University Hospital (NRH). This suggests there may be many people not accessing the specialist rehabilitation services they require.

**Conclusion:**

Data linkage between administrative and health datasets does not currently exist but offers huge potential for understanding the trauma and rehabilitation ecosystem in detail. This is required to better understand the impact of strategy and policy.

**Supplementary Information:**

The online version contains supplementary material available at 10.1186/s12913-023-09177-1.

## Introduction

Globally, road traffic collisions (RTCs) are a leading cause of death and serious injury with 1.35 million people dying each year and 20–50 million surviving with serious injury and disability [1]. Despite overwhelming evidence of the devastating health and socio-economic consequences of RTCs, until recently their impact has been overlooked in government strategy and policy [1]. In 2017, the Valletta Declaration on Road Safety established a target of halving the number of serious injuries in the EU by 2030 [2].

In Ireland, there has been a dramatic change in the road safety landscape over the last 25 years. In 1998, the first government strategy for road safety was published followed by the establishment of the Road Safety Authority (RSA) in 2006 as a statutory organization created by the Road Safety Authority Act, 2006 with the aim to reduce collisions, deaths, and injuries [3]. Since then, Ireland has improved from having the 11th lowest road mortality in the European Union (EU) in 2011, the year of the first Road Safety Performance Index (PIN) Report, to 2nd lowest in 2018 [4–6]. There have been 4 Road Safety Strategies with each one building on the progress and understanding provided by the previous strategies (1998–2002), (2004–2006), (2007–2012) with the objective of sustainably improving safety on Irish roads. The last strategy, ‘Closing the Gap’, covered the period 2013 to 2020 and in addition to continuing to reduce fatalities, several actions within the Strategy aimed to reduce the number of serious injuries.

### Data

A more recent development has been the National Office of Clinical Audit (NOCA) implementation of the Major Trauma Audit (MTA) in Ireland. The MTA is a clinically led audit established in 2013 using the well-established National Health Service (NHS) UK Trauma Audit and Research Network (TARN) [7]. TARN is the UK National Clinical Audit for traumatic injury and is the largest European Trauma Registry. The MTA captures data on patients of any age who sustain an injury resulting in any of the following: hospital admission > 72 h; intensive care or high dependency admission; transfer to a tertiary/specialist center and in-hospital death within 30 days [8]. Irish MTA reports have been published from 2014 to 2018 [9–12]. The reports comment on road trauma and head injuries with a section on severe TBI and cause (Chap. 3). In the reports a severe TBI is defined as Abbreviated Injury Scale (AIS)3 + and Glasgow Coma Scale (GCS) < 8/9 (depending on the report).

In the UK, it was recognized that there was very little information available about the rehabilitation needs of patients leaving the Major Trauma Centers (MTCs) or how well these needs were being met. To address this gap, the TARN dataset was linked to the UK Rehabilitation Outcomes Collaborative (UKROC) dataset, in the National Clinical Audit of Specialist Rehabilitation following Major Injury (NCASRI) [13]. Ireland thus far, does not have a Rehabilitation Outcomes Collaborative and therefore very little information is available about the rehabilitation needs of patients leaving trauma receiving hospitals or how well these needs are being met. Each part of the system currently collects its own measures which are siloed and fragmented with no common measure across the pathway.

There are also other sources of trauma and road traffic related data in Ireland including the Irish Hip Fracture Database, The Irish LongituDinal Study on Ageing (TILDA), the Road Safety Authority (RSA), the Health and Safety Authority (HSA), the Central Statistics Office (CSO), the Hospital Inpatient Enquiry (HIPE) system and An Garda Siochana (Irish police force) so the data landscape is complex. Data linkage between these administrative and health datasets does not currently exist but offers huge potential for understanding the trauma and rehabilitation ecosystem in detail.

### National Strategy

In addition, in 2018, the vision for a national trauma system was set out in the Report of the Trauma Steering Group, A Trauma System for Ireland [14]. This report introduced national standards for the delivery of trauma care with the aim of preventing unnecessary deaths, reducing disabilities, and significantly improving the patient’s chances of attaining the fullest possible recovery. With the implementation of the report, access to data across the continuum of care will be required to ensure the needs of all trauma patients are being met.

### Literature review

To understand how trauma systems and road safety policy impact on rehabilitation services, a desktop review of the literature was undertaken. This revealed several recent systematic reviews and meta-analysis exploring the impact of trauma systems on injury outcomes [15–17]. Most studies looked at mortality as the primary outcome measure. These reviews recommend that further research is required to properly evaluate the different components of trauma systems and non-fatal outcomes and explore the impact of system component interactions. Although there are many publications that look at the impact of elements of road safety strategy there were none that evaluated implementation of road safety strategy as a complex intervention in a complex system and most publications agreed on the need for further research exploring long term and patient-centered outcomes [18–22]. Also of note is that there is currently no recommended standardized dataset for measuring disability or health outcome across the continuum of trauma care.

### Aim

In the absence of an Irish Rehabilitation Outcomes Collaborative, and with the advent of the implementation of the new trauma report, this study aimed to review access to complex specialist rehabilitation for patients experiencing serious injury with the objectives of assessing if admissions with road traffic trauma related injury had changed over the course of the current road safety strategy and also how the numbers compared with those reported in the MTA reports. It was hypothesized that the implementation of the road safety strategies would result in lower admissions for rehabilitation.

## Methods

A retrospective review of paper healthcare records in a Complex Specialist Rehabilitation (CSR) Hospital. The National Rehabilitation University Hospital (NRH) is the only Complex Specialist Rehabilitation hospital in the Republic of Ireland. Specialist rehabilitation is the total active care of patients with complex disabilities by a multiprofessional team who have undergone recognized specialist training in rehabilitation, led /supported by a consultant trained and accredited in rehabilitation medicine. The NRH has a national remit and has five clinical programmes that provide CSR services to patients with an acquired complex disability as a result of an accident, illness, or injury. The programmes are: Acquired Brain Injury (ABI), Stroke Specialty, Spinal Cord System of Care (SCSC), Prosthetic, Orthotic and Limb Absence Rehabilitation (POLAR) and Paediatric Family-Centred Rehabilitation. It is fully publicly funded and is a tertiary referral hospital, accredited by the Commission for Accreditation of Rehabilitation Facilities. A broad range of data is recorded on admission, but the national data standard is Hospital In-Patient Enquiry (HIPE). HIPE is the principal source of national data on discharges from acute hospitals in Ireland and uses the Clinical coding scheme the International Classification of Diseases (ICD) 10.

All patients discharged from the in-patient rehabilitation service with an ICD 10 coded diagnosis of Transport accidents (V00 – V89.9) (Transport accident-related injury) from 2014 to 2018 were included. This period was selected as it corresponds with the data collection period of the MTA reports and most of the duration of the current road safety strategy. The International Classification of Diseases (ICD) is a globally used diagnostic tool for epidemiology, health management and clinical purposes. The ICD is maintained by the World Health Organization (WHO), which is the directing and coordinating authority for health within the United Nations System [23]. Inclusion criteria included Admission with RTC related injury between January 2014 and December 2018. Exclusion criteria: Readmissions due to issues related to the same RTC, and admission date before January 2014 or after December 2018.

Specific data gathered included age at first presentation; gender; admission clinical programme (Brain injury programme (BI); spinal cord injury programme (SCI), prosthetic orthotic and limb absence programme (POLAR), paediatric programme (Paeds); Main injury sustained; level of disability on admission (as measured by the modified Barthel Index), and year of admission.

The healthcare record review was performed in accordance with recommended best practice for the performance of retrospective chart review and medical record data abstraction [24–30]. Data was abstracted using a bespoke data abstraction instrument (password protected excel spreadsheet). Medical record abstraction is the manual process of collecting relevant information from a patient’s medical record and transcribing that information into discrete fields. Abstraction of the data was in accordance with the instrument devised and variables and definitions were agreed upon beforehand with a user manual developed (Appendix 1). The data abstractor (co-author) was trained on the variables and the data abstraction form. Following this review, the data abstractor coded 10 charts to check the method and the coded elements were verified by a co-researcher (lead author) to ensure accuracy. Any discrepancies or where clarification was required, were reviewed jointly, and discussed to clarify any issues. Weekly meetings were held to check coding quality and progress. Convenience sampling was used with all cases selected over a defined time. A simple coding manual was created. A numeric and written code was assigned for each potential answer to each heading. We adopted “9” as the universal code for missing data as recommended by the Health Services Executive (HSE) Healthcare Audit Criteria and Guidance [31].

In addition, the number of RTC related severe traumatic brain injury (TBI), spinal cord injury (SCI) and traumatic amputations were extracted from the MTA reports 2014–2018.

### Data analysis

Data analysis was conducted using statistical software package SPSS version 27. Univariate analyses exploring associations between injury type and demographics were performed using Fisher’s exact test and binary logistic regression models for brain injury and spinal cord injury were developed to explore the relative contributions of demographic factors. Statistical process control (SPC) was used to analyse variation. Anticipating a change in the number of admissions to the NRH as the result of implementation of road safety policy and strategy represents a complex interplay between policy and strategy, diverse patient casemix, fragmented data sources and imperfect hospital processes and therefore numbers of admissions often show some variation with repeated measurements, even when there is no change to the process. A control chart is a process improvement technique that can be used to monitor a variable during the implementation of new interventions or processes to assess if changes are followed by improvement. It is a graphic display of data against established control limits that reflect both maximum and minimum values. Control limits are set at three standard deviations from the mean and certain rules are applied to ascertain if a variation is expected (normal cause) or unexpected (special cause).

## Results

There were 338 cases identified in the 5-year study period. Of these, 179 cases did not meet the inclusion criteria due to readmissions with the sequelae of the same RTC, duplication of entries and miscoding (i.e., injury not RTC related). The total number of healthcare records analyzed systematically using the data abstraction instrument was 165.

**Fig. 1:**
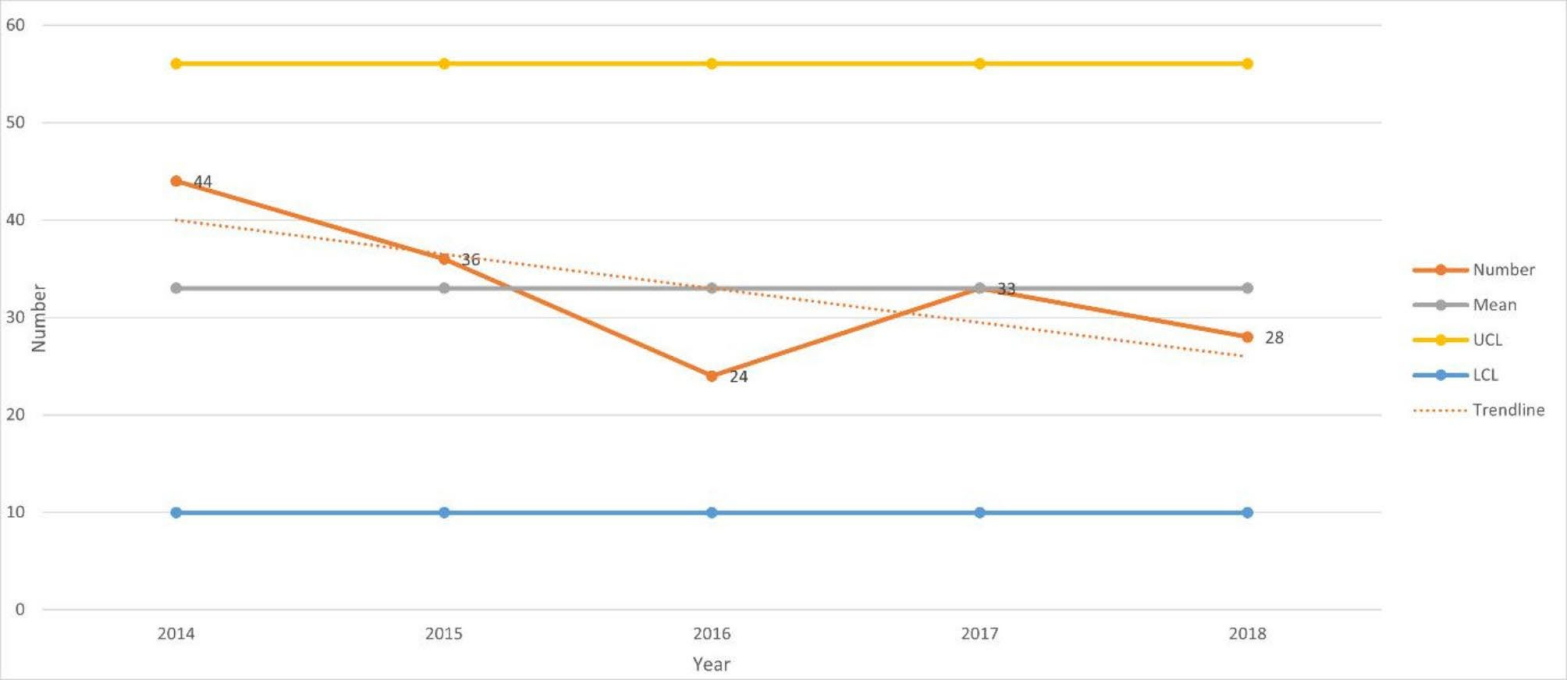
SPC of Numbers of RTC related admissions each year from 2014–2018

121 (73%) were male and 44 (27%) were female. 115 (72%) were under 40 years of age. 128 (78%) had traumatic brain injuries (TBI), 33 (20%) had traumatic spinal cord injuries (TSCI) and 4 (2.4%) had traumatic amputations (TA). There were no combined injuries. Figure 1 shows a Statistical process control (SPC) chart analysing the numbers of RTC related admissions to all programmes in the NRH each year from 2014 to 2018 showing the mean and upper control limit (UCL) and lower control limit (LCL) with trendline.

There was variation in the numbers over the 5 years studied with the trendline indication a reduction. However, when analysed in a statistical control chart, this was normal variation with no special cause variation. Special cause variation is present when the control chart of a process measure shows either plotted point(s) outside the control limits (UCL and LCL) or a non-random pattern of variation.

Traumatic amputations have been removed from subsequent analysis as the small sample size per year gives a risk of re-identification.

**Fig. 2:**
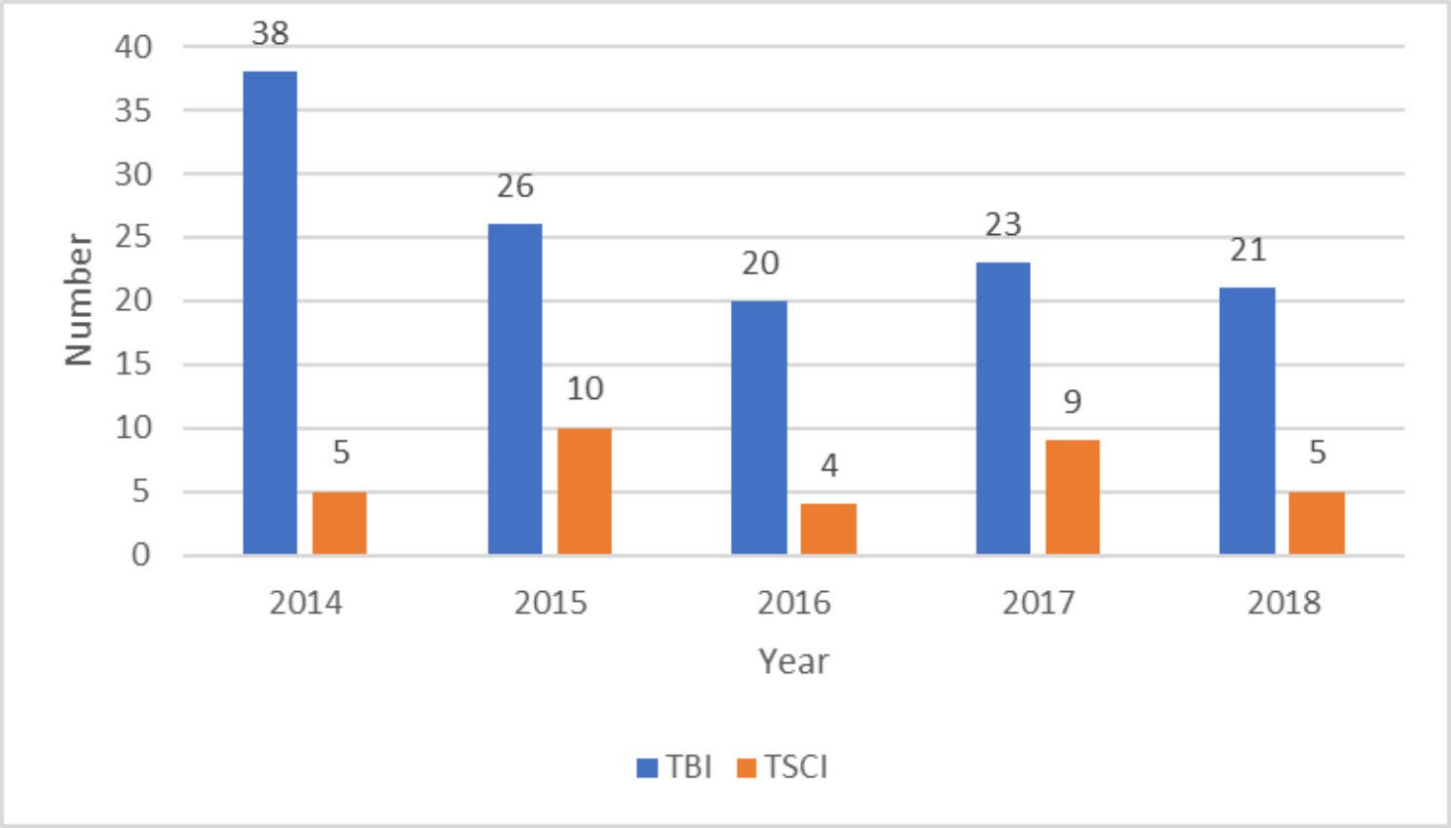
Numbers of Admissions Per Injury Type Per year

Figure 2 shows the numbers of admissions per injury type per year, traumatic brain injuries (TBI), and traumatic spinal cord injuries (TSCI).

Brain injury was the major injury type for both genders. Fisher’s exact test revealed that there was no significant association between injury type and gender categories (p = 0.273). In addition, brain injury was the major injury type for all age categories. Fisher–Freeman–Halton test revealed that there was a significant association between injury type and age categories (p = 0.024). Table 1 shows the breakdown of injury type by gender and Table 2 injury type by age category.

**Table 1:** Injury type by Gender

Injury type	Male n (%)	Female n (%)	Total n (%)
Brain injury	90 (74%)	38 (84%)	128 (77%)
Spinal injury	27 (22%)	6 (13%)	33 (20%)
Total	117 (100%)	44 (100%)	161 (100%)

**Table 2:** Injury type by age category

Injury type	< 18 yrs (%)	18–35 yrs n (%)	36–55 yrs n (%)	> 56 yrs n (%)	Total n (%)
Brain injury	36 (92%)	52 (78%)	30 (64%)	10 (83%)	128 (77%)
Spinal injury	3 (8%)	14 (21%)	15 (32%)	1 (8%)	33 (20%)
Total	39 (100%)	66 (100%)	45 (100%)	11 (100%)	161 (100%)

Two binary logistic regressions were performed to determine the effect of sex, age, and year of admission on brain injury and spinal injury (Tables 3 and 4 respectively). It was found that the age group 36–55 years had significantly less chance of getting brain injury compared to the younger age group (< 18 years) (Odds Ratio (OR) = 0.16, p = 0.009). Also, it was found that age group 36–55 years had a significantly higher chance of getting a spinal injury compared to the younger age group (< 18 years) (OR = 6.17, p = 0.009).

**Table 3:** Multivariate logistic regression model for brain injury

	Odds ratio	p	95% CI
Female (Ref. Male)	1.461	0.461	0.53–3.99
Age (Ref. <18 years)		0.043	-
18–35 years	0.304	0.081	0.08–1.16
36–55 years	0.162	0.009	0.04–0.63
56 + years	0.826	0.875	0.08–8.96
Year	0.826	0.18	0.63–1.09

**Table 4:** Multivariate logistic regression model for spinal injury

	Odds ratio	p	95% CI
Female (Ref. Male)	0.684	0.461	0.25–1.87
Age (Ref. <18 years)		0.043	-
18–35 years	3.284	0.081	0.86–12.51
36–55 years	6.179	0.009	1.59–24.07
56 + years	1.211	0.875	0.11–13.13
Year	1.211	0.18	0.92–1.60

All patients with a traumatic brain injury had a moderate to severe acquired disability on admission (Mean MBI 47; range 00–90). The level of disability for the other programmes was incomplete and therefore excluded.

When the MTA reports were analysed, the reports only detailed RTC related severe TBI (AIS3+, Glasgow Coma Sore < 9/8). RTC related Traumatic SCI and amputation numbers were not stated. In the 2016 report, the Glasgow outcome scale (a global scale for functional outcome) on discharge (GOSD) was reported but did not specifically look at RTC related injury. As we were unable to derive RTC related information from the disability measures, we chose to look at RTC related severe TBI.

**Fig. 3:**
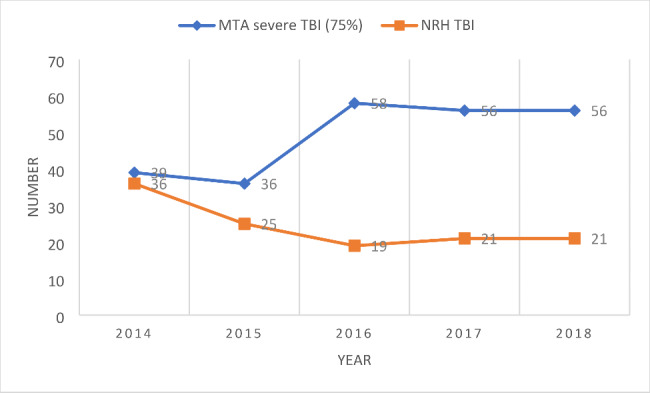
Comparison of NRH admissions with RTC related TBI and MTA RTC related severe TBI numbers

Figure 3 shows the numbers of admissions to the NRH with RTC related TBI and the number of severe TBI as reported in the MTA reports from 2014 to 2018.

## Discussion

Analysis of the data of inpatient admissions to a complex specialist rehabilitation hospital showed that road traffic collision related injuries were commoner in males (73%) and younger age groups (93% under 56 yrs). The most common injury was traumatic brain injury (77%) followed by traumatic spinal cord injury (20%).

### Admissions to NRH

There was an overall reduction in RTC related injuries over the 5 years studied but the numbers varied over each year. When analysed in a statistical control chart, this was normal variation with no special cause variation. Special cause variation is present when the control chart of a process measure shows either plotted point(s) outside the control limits or a non-random pattern of variation and would be expected if an intervention was having an impact. This suggests that there has been no impact of any intervention such as road safety strategy, on admissions with road traffic related injuries over the time period studied.

### MTA data

The MTA reports only reported on 1 occasion on TBI related disability measures (GOSD) and did not look specifically at RTC related TBIs. The MTA reports define severe head injury patients by Abbreviated Injury Scale (AIS) classification (AIS ≥ 3) and GCS < 9/8, depending on the report. The NRH as a complex specialist rehabilitation hospital only admits patents with category A, complex care needs as determined by the Rehabilitation Complexity Scale—E or moderate to severe disability (MBI < 60). [32] 2 recent EU projects (SafetyCube [33] and Study on serious road traffic injuries in the EU [34]) have shown that 75% of individuals with severe RTC related injuries have long term difficulties. Therefore, although not directly comparable and acknowledging that GCS is a poor predictor of outcome, the AIS together with the GCS does give an indication of severity of injury. Therefore, it is reasonable to assume that many of these patients will have complex disabilities as a consequence of their TBI. Using the data collected in our review, and taking 75% of the numbers of severe TBI, there is a large discrepancy between the number of severe TBIs reported in the MTA reports and the numbers of RTC related TBI being admitted for complex specialist rehabilitation (Figure 3).

### Access to rehabilitation

The National Rehabilitation University Hospital is the only CSR facility in Ireland and unless these patients are being transferred to other countries, this means patients may not be accessing the level of rehabilitation services they require. If this is the case, this is not in keeping with article 26 of the United Nations Convention on the Rights of Persons with Disabilities (UNCRPD) [35] and is not in keeping with the Rehabilitation Medicine Model of Care or the National Policy and Strategy for the Provision of Neuro-Rehabilitation Services in Ireland [36, 37]. In addition, a recent (unpublished) audit of delayed transfers of care by the HSE Rehabilitation Medicine Programme showed that many of these patients remain in acute hospitals for protracted periods of time.

Rehabilitation services are recognized internationally as the “missing link” between hospital and community services but have traditionally been seen as a “nice to have” rather than an essential component of a trauma system of care. However, integration of rehabilitation services into the continuum of care within and beyond acute hospitals will accelerate discharge planning, reduce disability and cost of care [38, 39]. As stated in the trauma report, a strong focus on comprehensive, patient-centred rehabilitation services is required, with early assessment of rehabilitation need, as well as enhanced acute, post-acute, regional and community rehabilitation, to enable patients to achieve their maximum functional potential.

### Practical implications

Key to an effective trauma system and road safety strategy is excellent knowledge mobilization, enabling research, care management, and performance improvement. However, as has been shown by this review, although there are several existing databases and information management systems along the trauma pathway, they are fragmented and there are significant gaps (particularly from a rehabilitation point of view) through which patients may be falling.

In order to understand the impact of any road safety strategy or implementation of the trauma report, addressing this fragmentation and tackling these knowledge gaps are essential if we are to successfully evaluate the impact of these policies and strategies.

### Limitations

The sample size was small which may hinder the generalization of the study findings The study did not collect data for all the years the current RSA strategy has been in place as the MTA reports only started in 2014. The NRH admission numbers reflect individuals with complex care needs because of RTC related injury and the MTA data records injury severity as measured by the AIS and GCS and not disability. Also, MTA data has not got 100% coverage so not all RTC related serious injury will be recorded.

## Conclusion

The number of admissions to a National Rehabilitation Hospital with Road traffic collision related injury was variable over the course of the 5 years of review but demonstrated normal cause variation and not special cause variation. In addition the MTA data showed that numbers of severe TBI were static. Taken together, this data would suggest that road safety strategies are not impacting on RTC related serious injury. The current fragmented nature of trauma data may have affected the outcome of the study. The NRH data and the MTA data show that there may be large numbers of patients not accessing the complex specialist rehabilitation services they may require. Linking data between MTA and NRH admission information may be an effective way to better understand the pathways of care for these patients and to study more accurately the impact of RSA strategy and trauma policy. Ultimately, the evaluation of the success of road safety and trauma policy and strategy will be dependent on the quality of the underlying data that reflects the continuum of people surviving with life altering injuries. There is much work to be done in making this a reality. Data linkage between these administrative and health datasets does not currently exist but offers huge potential for understanding the trauma and rehabilitation ecosystem in detail.

## Electronic supplementary material

Below is the link to the electronic supplementary material.

Supplementary Material 1

## Data Availability

The datasets generated and/or analysed during the current study are not publicly available due the constraints of ethical approval but are available from the corresponding author on reasonable request.
