# Supplementary material for: The impact of road safety strategy and policy on admissions to a national rehabilitation hospital; a 5-year retrospective review and reflection on trauma data
Source: BMC Health Serv Res. 2023 Feb 21;23:175. doi: 10.1186/s12913-023-09177-1 (PMC9942335; doi:10.1186/s12913-023-09177-1)
Supplement: Supplementary file 1 — Supplementary Material 1 [file 12913_2023_9177_MOESM1_ESM.docx]

Retrospective chart review user manual

# **Title: The Impact of Road Safety Strategy and Policy on admissions to A National Rehabilitation Hospital; A 5-Year Retrospective Review**

This study aims to review access to complex specialist rehabilitation for patients experiencing serious injury with the objectives of assessing if admissions with road traffic trauma related injury has changed over time in response to national road safety strategy and also how do the numbers compare with those figures reported in the MTA reports.

This is a type of research methodology referred to as a retrospective chart review.

The coders have provided you with an excel spreadsheet of all admissions with a ICD 10 coded diagnosis of Transport accidents (V00 – V89.9) (Transport accident-related injury) from 2014-2018.

Each day, at 9am, the healthcare record team will provide you with 10 charts to code from healthcare records. You will sign for the charts in the tracking record and you are responsible for the security of the charts while they are in your care.

Please return the charts to healthcare records by 4.30pm each day and sign the tracking sheet.

Please inform healthcare records by midday of the 10 charts you wish to code the following day.

## Data collection:

You will have been inducted in the format of the healthcare records and how to systematically review them. Most information will be located in the discharge summary found at the front of the chart in the correspondence section.

If the information is not in the correspondence section, then go to the Clinical notes section and locate the most recent admission proforma.

First, check the MRN against the list.

The following variables are to be collected:

1. **Initials**: First name and Last name initials eg John Paul Smith (JS)
2. **Gender**: M=1; F=2; Unspecified: 0
3. **Age on admission**: Calculate age on **first** admission with RTC related injury
4. **RTC related injury** 1=Yes 2=No If you are not sure, put the chart aside and discuss with Prof Carroll
5. **Type of Injury sustained**:

TBI: Traumatic brain injury

TSCI: Traumatic spinal cord injury

TA: Traumatic amputation

Comb: combination of above and specify which

Other:

1. **Clinical Programme**:

Which programme was the patient admitted to:

Brain injury programme (BIP)

Spinal Programme (SP)

Prosthetic, orthotic or limb absence programme (POLAR)

Paediatric Family-Centred Rehabilitation Programme (PFCRP)

1. **Year of admission:** Year of first admission with RTC related injury (2014-2018)
2. **Admission Outcome measure:**

Modified Barthel Index.

This will be recorded in the discharge summary located in the correspondence section at the front of the chart.

If not, then locate the outcomes section of the record.

If it is not in the outcomes section then locate the nursing section.

If it is not in any of these sections them mark as missing ‘9’, set the record aside and discuss with Prof Carroll.

**If you are in doubt about any of these variables, please contact Prof Carroll.**
